# Basosquamous Carcinoma: A Single Centre Clinicopathological Evaluation and Proposal of an Evidence-Based Protocol

**DOI:** 10.1155/2018/6061395

**Published:** 2018-06-03

**Authors:** Jordan W. Oldbury, Richard A. J. Wain, Sameera Abas, Christopher M. Dobson, Srinivasan S. Iyer

**Affiliations:** ^1^Department of Plastic & Reconstructive Surgery, Royal Preston Hospital, Lancashire Teaching Hospitals NHS Foundation Trust, Sharoe Green Lane, Preston, Lancashire PR2 9HT, UK; ^2^Department of Dermatology, Royal Preston Hospital, Lancashire Teaching Hospitals NHS Foundation Trust, Sharoe Green Lane, Preston, Lancashire PR2 9HT, UK

## Abstract

Basosquamous carcinoma (BSC) is an uncommon skin malignancy with significant invasive and metastatic potential. There are currently no clear management guidelines. This study evaluates the management and outcomes of patients diagnosed with BSC over a 7-year period. We present an evidence-based unit protocol for the management of BSC. All patients treated for BSC between 2009 and 2015 were reviewed. Data collected included patient demographics, tumour-specific information, management strategy, presence of recurrence or metastasis, and details of follow-up. 74 patients were identified, making this one of the largest cohorts of BSC patients reported. Mean age at diagnosis was 75.4 years, with a male:female ratio of 1.6:1. The most common tumour site was the head and neck (n=43, 58.1%). All tumours were graded at pT1 (n=51) or pT2 (n=23). Inadequate excision occurred in 17 patients (23%). Mean excision margins were >4mm peripherally and deep. Inadequately excised BSCs were further treated with wide local excision (n=6) or radiotherapy (n=5), or both (n=1). There were no cases of local recurrence or metastatic disease. This study demonstrates a cohort of patients with BSCs that appear less aggressive than previously reported. Current management with surgical excision appears to produce adequate results. However, an evidence-based guideline is still lacking.

## 1. Introduction

Basosquamous carcinoma (BSC) is a rare form of skin cancer currently representing approximately 2% of all nonmelanoma skin malignancies [1–5]. The majority of cases are found in the head and neck region with preponderance for older Caucasian males [2, 6, 7]. Synonymously labelled metatypical basal cell carcinoma (BCC), BSCs are almost invariably clinically indistinguishable from BCCs but are said to be more aggressive and invasive than both BCCs and squamous cell carcinomas (SCCs) alone, with higher rates of recurrence (reported as up to 45%) and metastasis (approx. 5-10%) [2, 4, 5, 8].

Since its original description in 1910, there has been debate about the terminology, definition, and subsequent management of BSC [3, 9–11]. These tumours are of unpredictable behaviour and mixed morphology, demonstrating features of both BCC and SCC with, or without, a transition zone between the two cell types [8, 12]. Initially, it was suggested that BSCs represent “collision” tumours, whereby SCC and BCC tumours independently develop apposed to one another [5]. However, given the histological features of BSCs and their pluripotency, it has more recently led to the development of the squamatisation theory, which suggests that BSCs are actually basal cell carcinomas that undergo squamous differentiation [10]. This is supported by the definition provided by the World Health Organisation (WHO) [13], who have classified BSC as “basal cell carcinomas that are associated with squamous differentiation”.

It is virtually impossible to clinically diagnose a BSC; however, the aid of dermoscopy alongside suspicious clinical behaviour might be beneficial. It has been shown that there are certain dermoscopic features which increase suspicion for BSC and enhance diagnostic accuracy [14], although histological confirmation by way of a punch or incision biopsy is essential prior to confirming diagnosis and initiating the correct treatment pathway. It should be noted, however, that easily excisable lesions or lesions with dermoscopic features suggestive of SCC should be excised as per the European Dermatology Forum (EDF) guidelines or the British Association of Dermatology (BAD) guidelines, dependent on country of practice (available from [15, 16]).

Whilst the ideal management strategy for BSC has not yet been established, the principal method apparent in the literature is surgical excision. Mohs micrographic surgery (MMS) has been shown to reduce recurrence rates when compared to standard excision and is the gold standard of treatment [1, 3, 8]; however, the inability to clinically distinguish BSC from BCC preoperatively, the minimal availability of MMS in the UK, and the current practice of excision biopsy of primary cutaneous lesions at presentation make patient selection challenging. Other techniques such as radiotherapy, laser ablation, cryotherapy, and chemotherapy have also been utilised in some cases, although mainly in stage 4 disease and without any significant evidence in literature [17].

It has been suggested that BSC requires a different approach with respect to treatment and follow-up than for BCC [18]. However, specific guidelines do not currently exist for management of BSC, and, given the inability to clinically distinguish BSC from BCC, histological confirmation is essential. We evaluated a cohort of patients with BSC over a 7-year period to determine the tumour characteristics, management strategies, follow-up regimens, recurrence rates, and incidence of metastasis. Data was compared to that available in the current literature, and an evidence-based unit protocol was devised.

## 2. Materials and Methods

This is a retrospective, monocentric study involving all patients treated for basosquamous carcinoma between 2009 and 2015 by our unit. Patients were identified using the institutional database in the Department of Pathology. Both clinical and histological characteristics reported at diagnosis were analysed. Exclusion criteria comprised those patients with an incomplete data set and patients lost to follow-up.

Clinical data was acquired from patient records and multidisciplinary team outcomes over several clinical sites. Variables including gender, age at diagnosis, primary tumour site, excision margins, need for wider excision or radiotherapy, follow-up regimen, and presence of recurrence and/or metastasis were recorded.

All histological specimens were excised as per BCC protocol and examined primarily by a dermatopathologist using haematoxylin and eosin (H+E) staining as a minimum. A small proportion of specimens were subsequently subjected to immunohistochemistry analysis using an anti-human epithelial antigen monoclonal antibody BerEP4, where the diagnosis was less clear. Diagnosis was made in accordance with the Royal College of Pathologists Standards for Histological Reporting of Primary Cutaneous BCC guidelines (available from [19]), and all diagnoses were reexamined by a specialist dermatopathologist through the specialist skin cancer multidisciplinary team to reconfirm diagnosis and establish a management plan. Excision margins, the presence of perineural or lymphovascular invasion, AJCC pT stage, and the status of reexcision specimens were also recorded when data was available.

Using a combination of the data collected, the results of this study, and current practices reported in recent literature, we have devised a new local protocol for the treatment and follow-up of this uncommon skin malignancy.

## 3. Results

**Figure 1:**
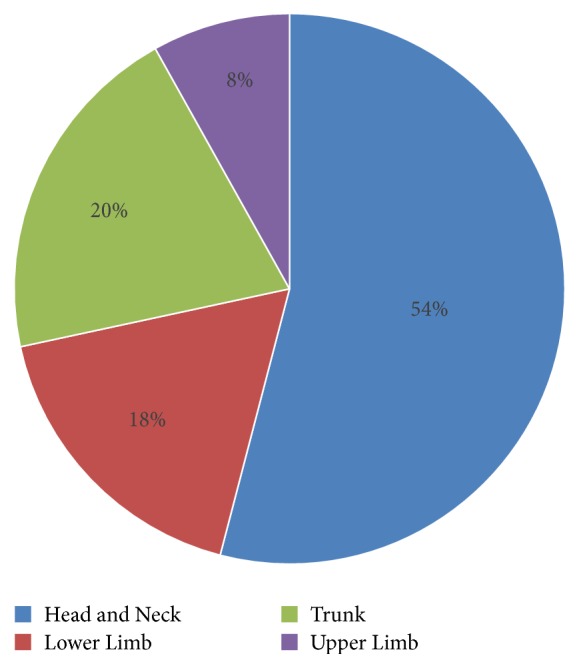
The most common area for BSC presentation was in the head and neck region (54% of cases), followed by the trunk.

There were 74 patients included in the study with a male:female ratio of 1.6:1 and a mean age at diagnosis of 75.4 years (range 44-95 years). There were no re-referrals following discharge. There were no deaths during the follow-up period. The most common tumour site was the head and neck (58.1% (n=43)), followed by the trunk (20.3% (n=15)), lower limb (17.6% (n=13)), and upper limb (8.1% (n=6)).  Figure 1 represents this data graphically. All lesions were pT1 (n=51) or pT2 (n=23), with 1 lesion displaying lymphovascular invasion and 4 lesions with (5.5%) perineural invasion. Mean excision margins were 4.5mm peripherally (range <1–13mm, SD 2.6mm) and 4.0 mm deep (range <1mm-16mm, SD 3.5mm). However, of the 73 patients for which we have data, the inadequate excision rate (defined as resection margins <1mm or involved margins) was relatively high (23%, n=17) with 59% (n=10) of these occurring at the deep margin, 29% (n=5) at both peripheral and deep margins, and 12% (n=2) at the peripheral margins alone.

**Figure 2:**
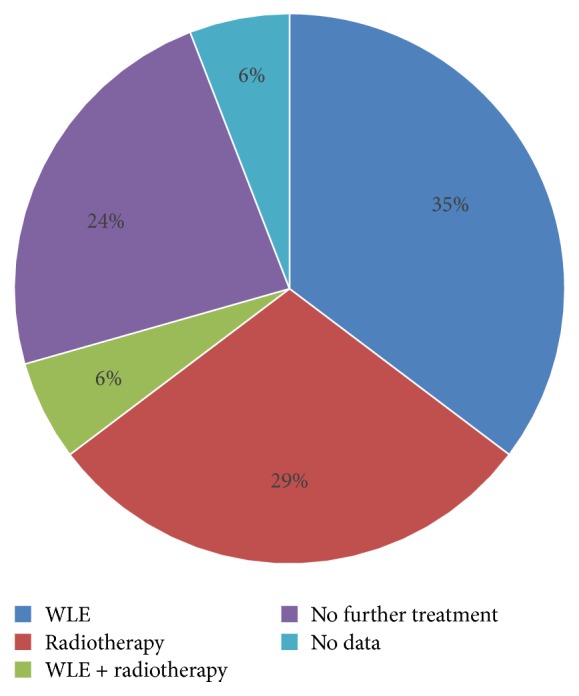
Follow-up and further treatment amongst patients with inadequately excised BCCs were heterogenous and varied.

Further treatment of inadequately excised BSCs (defined as margins ≤1mm) involved either wide local excision (WLE) (n=6), radiotherapy (n=5), or both WLE and radiotherapy (n=1). No further treatment was given to four patients due to consultant decision to observe, and there was no data available for one patient (see Figure 2).

Only one of the WLE specimens showed evidence of residual malignancy. The duration of follow-up varied widely (5-60 months), with a mean length of follow-up of 20.2 months. Eight patients are still under review. Indeed, the interval between routine follow-up appointments following diagnosis also varied (3-6 months), with a mean of 4.4 months. There were no cases of local recurrence and no patients with metastatic disease at the time that the study was carried out.

## 4. Discussion

Basosquamous carcinoma is an uncommon, aggressive malignancy for which current literature has little consensus and is seen by some as a distinct pathology to both BCC and SCC [20]. However, there is long-standing discord over where BSC falls in the nonmelanoma skin cancer spectrum, and current consensus leans towards BSC representing a subtype of BCC. Early diagnosis and identification are key to optimal clinical outcome, given the reported aggressive nature of the disease and high risk of recurrence and metastasis. Due to experience and research with both BCCs and SCCs, surgical excision is currently the first-line treatment option, but a significant role has been demonstrated for radiotherapy and Mohs' micrographic surgery [5, 11, 21–24]. To date, there is no standardised treatment protocol for the management of basosquamous carcinoma [25].

The current study has demonstrated that primary excision of pT1 and pT2 BSC tumours can lead to good clinical outcome, with or without the need for further excision, with none of the 74 patients involved in this study developing metastasis or recurrence of disease. This is in line with Kececi* et al.*, who demonstrated no metastasis and a recurrence rate of only 4% in a series of 35 patients [7]; however, these figures are lower than other reported studies (see Table 1).

Previous reports demonstrate a strong male preponderance and an increased occurrence in the head and neck regions. Our study reflects these findings, albeit not as convincingly as the previous studies [4, 5, 7, 29]. These demographics are important to consider on clinical diagnosis of a skin malignancy given that a predisposition to development on the face leads to increased difficulty in resection and reconstruction, problems achieving adequate surgical margins, and issues in providing a good aesthetic outcome.

This is one of the largest reported studies involving only BSCs to date, and our findings are comparable with many previous studies. However, despite a large proportion of previous authors reporting high recurrence and metastasis rates, at the time that this study was carried out, we had not recorded a single patient within this cohort with recurrence or metastasis. We did not identify any patients with pT3 or pT4 tumours, which could account somewhat for this observation. Wermker* et al*. [2] reported a cohort of 89 patients, 18 patients of which were found to have pT3 or pT4 tumours. Of these patients, three patients (16.7%) developed lymph node metastases, whereas only 2/71 (2.8%) of those patients with pT1 or pT2 tumours developed lymph node metastases. Furthermore, the authors reported no change in progression-free survival at 18 months of follow-up in those diagnosed with pT1 and pT2 tumours. The findings of the current study, as well as those of Wermker* et al.*, go some way to explaining how we may have identified a cohort wherein recurrence and metastasis are unlikely to occur.

For the 71 patients for which we could collect data regarding subsequent treatment following the primary surgical excision, 17 patients had inadequately excised lesions, of whom nine (11.3%) patients had involved surgical margins. In this cohort, subsequent treatment varied widely. Previous reports have demonstrated positive margins in more than 30% of cases following primary excision, usually presumed due to the microscopic invasiveness of the disease, and this is well documented to be a significant risk factor for recurrence of disease [4, 7, 25, 30]. The most likely reason for this low incidence is the generally low clinical staging of disease, but this has yet to be tested and evaluated through further research.

Out of the 73 patients for which we had data in this cohort, four (5.5%) patients had histological evidence of perineural invasion, one of the significant risk factors for recurrence [1, 4]. Although the incidence in this cohort falls within previously reported incidence of perineural invasion (0-7.9%), it is likely that no recurrence has been seen in the current study due to the absolute number of BSCs demonstrating perineural invasion being small [1, 28]. It is possible that a larger cohort would demonstrate recurrence if the proportion of specimens demonstrating perineural invasion stayed the same.

### 4.1. Metastasis

Despite being relatively rare, the concerning aspect of BSC is its ability to metastasise. The treatment of metastatic BSC is complex with poor outcomes, with one study reporting an average life expectancy of 1.6 years at diagnosis [31]. This, however, appears to considerably underestimate the survival rate, with a further study reporting an overall survival rate in patients with significantly advanced diseased as 54% (95% CI, 19%-80%) [17].

The prevalence of metastasis in BSC is known to be higher than in BCC and SCC. In a case series of 1000 consecutive tumours treated with MMS, the authors report a significantly increased prevalence of metastasis in BSC (n=27; 2 (7.4%) with pulmonary metastasis) compared with SCC (n=228; 2 (0.87%) with pulmonary metastasis) [5]. This is supported by a case series of 28 patients, in which nine patients developed recurrent disease, five patients developed disease in the lymph nodes, and one developed pulmonary metastasis [4]. Wermker* et al*. [2] reported five lymph node metastases in a series of 89 patients, with progression-free survival time averaging 16.1 months (range: 8.1-24.1 months); of these, two patients had pT1/pT2 tumours, with the remaining three having pT3/pT4 disease. BCC is known to very rarely metastasise [20, 32].

There is no difference in the clinical characteristics between BSC and other types of BCC (with the exceptions of sclerosing BCC and pigmented BCC), but there is a significant difference in the histological phenotypes. This could suggest a difference in the genotypes of BSC compared with other subtypes of BCC, suggesting a possible explanation for the difference in rates of metastasis.

### 4.2. Recurrence

Previous studies have reported a wide range of local recurrence rates of between 4 and 47.1% (as reviewed by Kececi* et al*., and Volkenstein* et al.*), and the risk of recurrence has been found to be increased by male gender, positive resection margins, and perineural and lymphatic invasion [5, 7, 11]. Clearly, a reduction in the rate of recurrence would lead to better clinical outcomes as well as reducing the rate of metastasis, and so care must be taken to ensure adequate surgical excision margins.

### 4.3. Mohs' Micrographic Surgery

Bowman* et al*. demonstrated that the number of stages of Mohs' micrographic surgery (MMS) required to achieve tumour-free margins in BSC was not significantly different to the number needed to treat SCC and BCC [5]. An initial randomised controlled trial studying the use of MMS for facial basal cell carcinoma in 408 patients with 30 months of follow-up demonstrated that the use of MMS slightly lowered the recurrence rates of BCCs, but this decrease did not reach statistical significance [33]. Subsequently, the same authors reanalysed the same population of patients and demonstrated a significantly lower rate of recurrence following MMS compared with surgical excision at 10 years of follow-up [24]. This robust study provided significant evidence for the use of MMS in nonmelanoma skin cancers; however, as the reported study did not look specifically at BSC, the results should be interpreted with caution in the context of the current study.

Whilst evidence suggests MMS may be the treatment of choice for these lesions with respect to recurrence rates and completeness of excision, the inability to clinically distinguish BSC from BCC preoperatively makes selection for MMS a technical and logistical challenge [1, 5]. Further considerations for the use of MMS for the treatment of BSC include identifying sources of funding and a much higher cost associated with MMS compared with surgical excision, the effect of a time-consuming surgery and its tolerability in an inherently elderly population, and the lack of national guidance in the treatment of BSC.

In this study, we demonstrated a perineural invasion rate of approximately 5.5%; this figure supports a potential role for MMS in the treatment of these skin lesions given that MMS ensures margin control in the excision of more aggressive tumours [34] and reduces recurrence rates in other cutaneous malignant lesions [24]. Currently, our service does not offer MMS for patients presenting with BCCs who are subsequently histologically diagnosed with BSCs, but this would be a valuable treatment option in the future when the service becomes available. The significant benefit of MMS is that the operating surgeon can more accurately secure disease-free margin, and the relatively high inadequate excision rate seen in this study would be significantly reduced.

### 4.4. Use of Radiotherapy

In our series, 6 (8.2%) patients were treated with adjuvant radiotherapy following primary surgical excision; however, there are currently no reports in the literature studying the use of radiotherapy in BSC. Cure rates of up to 91-93% have been seen with radiotherapy alone in treating BCC and that an approach utilising both surgery and radiotherapy together demonstrate cure rates of approximately 95% [35]. Given the close histological appearances between BCC and BSC, it would suggest that, in appropriate patient groups, treatment with radiotherapy either alone or in combination with surgery may be an appropriate option for the management of BSC if standard surgical excision or MMS is not possible [31].

### 4.5. Limitations

As demonstrated, our cohort of patients comprised only pT1 and pT2 cancers; therefore, reported rates of recurrence and metastasis would not accurately reflect the true recurrence/metastasis rate for the entire BSC population. We have also found that follow-up practices vary greatly, with variation between 5 and 60 months; this is a symptom of the lack of national guidance for the treatment of BSC. Additionally, the data used in this study is retrospective, heavily reliant on the availability of recorded data, and spread across multiple clinical sites. The findings of this study, therefore, must be interpreted with caution.

Given the methods by which the patients were identified in this study, it is possible, however unlikely, that the diagnosis of BSC has been missed in the full population of BCC patients in the time period identified. It should be noted that the number of BCCs excised in this time period is significant, and therefore subsequent reanalysis of all BCC specimens to identify any missed BSC diagnoses is not practical. All cases of BSC diagnosed in the time period were included. In cases where recurrence or metastasis was identified in patients originally diagnosed with BCC/SCC, the primary lesion was reexamined by an independent pathologist; no further cases of BSC were identified in this process. There were no cases of metastatic BCC at our centre in this time period.

It remains unclear whether BSC develops as a distinct clinical entity or whether BCC lesions undergo squamatisation. In this study, only those specimens in which the diagnosis was not clear on H+E staining were subjected to further analysis using BerEP4 immunohistochemistry staining. This anti-human epithelial antigen antibody labels the basaloid component of basal cell carcinoma, which in turn allows identification of a transition zone within the BSC itself [29]. When the transition zone is not present, the staining suggests the presence of collision tumours, as SCCs are typically negative for BerEP4 [11]; however, the lack of transition zone is not diagnostic [7]. Mougel* et al*. demonstrated in a 15 case series that 20% of those lesions initially labelled as basosquamous carcinoma were eventually diagnosed as SCC [28]. This was supported by a case series of 178 patients, wherein the initial diagnosis of BSC was only correctly given in 13.7% of cases [1]. Farmer* et al*. reanalysed cases of metastatic BCC and demonstrated that 80% of malignancies initially thought to be BCC had basosquamous differentiation on histology of metastases [31]. Given the retrospective nature of our evaluation of histological specimens, it is possible that some of the cases initially labelled as BSC were actually SCC and that some of the malignancies labelled as BCC and excluded were actually BSC. Future prospective studies should include immunohistological staining for BerEP4 as a standard to facilitate diagnosis of BSC [29].

### 4.6. Proposed Local Protocol

Whilst none of the available published studies have large numbers or prospective data collection [3], there are trends between the studies that can guide an approach to management and follow-up. In particular, the study by Wermker* et al*. [2] was conducted in a robust fashion and has shown a clear relationship between progression-free survival (PFS) and tumour stage, as well as between PFS and maximal vertical invasion of the tumour. Given the current lack of guidance available specifically for BSC, it would seem reasonable to base a unit BSC management protocol on the best available literature and the results of the current study. Our suggested unit protocol is demonstrated in Box 1.

## 5. Conclusion

In this patient group, BSCs appear less aggressive and less likely to recur or metastasise than those stated in the literature despite incomplete excision rates being comparable with other reported studies. Current management including surgical excision, with or without radiotherapy, appears to be the best method of management, with future consideration of inclusion of Mohs' micrographic surgery in the treatment protocol when the service becomes available. As Wermker* et al*. [2] noted, further prospective studies are needed, and practical, evidence-based protocols are pending the findings of these studies. Follow-up regimens locally are variable and require standardisation in the form of a guideline based on this study and the best available current literature. This is certainly necessary at a local level but perhaps could be used more widely. We propose a BSC management protocol for use in our unit in an attempt to standardise management and ensure improved quality care in this potentially challenging patient group.

## Figures and Tables

**Box 1:**
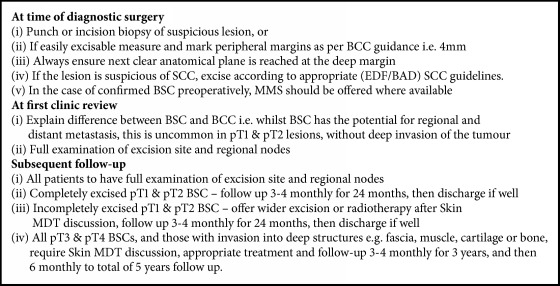
Using the data presented in this study in conjunction with published reports, a proposed unit protocol has been designed to guide treatment and follow-up for patients diagnosed with BSC.

**Table 1:** A comparison of studies of the recurrence and metastatic rates of BSC following excision. NR = not recorded. *∗* = only 25/35 patients available for follow-up, hence the higher percentage.

**Study**	**No. of cases**	**Recurrence**	**LN/Distant Metastasis**
**Current study**	74	0	0

**Schuller *et al*., 1979 [26]**	33	4 (12%)	NR

**Martin *et al*., 2000 [4]**	28	9 (32%)	5 (18%)

**Bowman *et al*., 2003 [5]**	27	NR	2 (7.4%)

**Leibovitch *et al*., 2005 [1]**	98	4 (4.1%)	0

**Skaria, 2010 [27]**	56	5 (8.9%)	NR

**Mougel *et al*., 2012 [28]**	12	0	0

**Betti *et al*., 2013 [12]**	35	2 (5.7%)	1 (2.9%)

**Kececi *et al*., 2014 [7]**	35	1 (4%*∗*)	0

## Data Availability

Readers may access the raw data underlying the study by contacting the corresponding authors using the contact details provided.
